# Characterizing the neurotranscriptomic states in alternative stress coping styles

**DOI:** 10.1186/s12864-015-1626-x

**Published:** 2015-06-02

**Authors:** Ryan Y. Wong, Melissa S. Lamm, John Godwin

**Affiliations:** Department of Biological Sciences, W.M. Keck Center for Behavioral Biology, North Carolina State University, Box 7614, Raleigh, NC 27695-7614 USA; Current Address: Department of Biology, University of Nebraska at Omaha, Omaha, NE 68182 USA

**Keywords:** Stress, Coping style, Proactive, Reactive, Anxiety, Brain, Danio rerio, Transcriptome, RNA-sequencing, Gene coexpression network

## Abstract

**Background:**

Animals experience stress in many contexts and often successfully cope. Individuals exhibiting the proactive versus reactive stress coping styles display qualitatively different behavioral and neuroendocrine responses to stressors. The predisposition to exhibiting a particular coping style is due to genetic and environmental factors. In this study we explore the neurotranscriptomic and gene network biases that are associated with differences between zebrafish (*Danio rerio*) lines selected for proactive and reactive coping styles and reared in a common garden environment.

**Results:**

Using RNA-sequencing we quantified the basal transcriptomes from the brains of wild-derived zebrafish lines selectively bred to exhibit the proactive or reactive stress coping style. We identified 1953 genes that differed in baseline gene expression levels. Weighted gene coexpression network analyses identified one gene module associated with line differences. Together with our previous pharmacological experiment, we identified a core set of 62 genes associated with line differences. Gene ontology analyses reveal that many of these core genes are implicated in neurometabolism (e.g. organic acid biosynthetic and fatty acid metabolic processes).

**Conclusions:**

Our results show that proactive and reactive stress coping individuals display distinct basal neurotranscriptomic states. Differences in baseline expression of select genes or regulation of specific gene modules are linked to the magnitude of the behavioral response and the display of a coping style, respectively. Our results expand the molecular mechanisms of stress coping from one focused on the neurotransmitter systems to a more complex system that involves an organism’s capability to handle neurometabolic loads and allows for comparisons with other animal taxa to uncover potential conserved mechanisms.

**Electronic supplementary material:**

The online version of this article (doi:10.1186/s12864-015-1626-x) contains supplementary material, which is available to authorized users.

## Background

Animals experience stress in a variety of naturalistic and artificial contexts and often successfully cope. The stress response and related coping mechanisms are essential and adaptive to an individual. Organisms that are unable to adequately cope with stressors often do not survive in the wild or in the case with humans, may be diagnosed with a mental health disorder [1-4]. An animal or human’s ability to cope with stress is influenced by genetic and environmental factors. While the neuroendocrine mechanisms of the stress response have been extensively studied, it is also important to understand the molecular mechanisms for coping with stress. In this study we characterize the neurogenomic mechanisms associated with differences in stress coping using wild-derived zebrafish lines selectively bred to exhibit variation in coping with stress.

Across a diverse range of animal taxa, two qualitatively different stress coping styles have been repeatedly documented: proactive and reactive coping styles [1,2]. Individuals with a proactive coping style are characterized by relying on a feed-forward memory process (i.e. actively exploring novel or anxiety-inducing environments as if the organism has previously encountered the scenario), possess low behavioral flexibility, and exhibit a relatively low glucocorticoid stress response [1]. In contrast, individuals displaying a reactive coping style will often wait to gather additional environmental information before responding. Reactive coping style individuals also tend to have high behavioral flexibility and will have a higher glucocorticoid stress response [1]. Both coping styles are adaptive responses to challenges in the environment, and theory predicts they will be maintained in a population due to different fitness optima in variable environments [2,3]. While individual variation exists for the magnitude of the responses within each coping style, mechanisms underlying this variation are not well understood (but see [5,6]).

Although an established model for developmental biology and toxicology [7,8], the zebrafish (*Danio rerio*) is only recently emerging as a valuable translational model system to study human health [9-17]. Zebrafish and mammals share a high degree of similarity in their genomes and neuroanatomy [15,18]. Teleost fishes also have a well-characterized stress response, and zebrafish can be quickly screened for and bred to exhibit differences in trait anxiety-like responses [11,19-22]. For example, our wild-derived HSB (High Stationary Behavior) and LSB (Low Stationary Behavior) lines of zebrafish exhibit characteristics of a reactive and proactive coping style, respectively, that is consistent across both time and contexts [19]. Importantly the behavioral, neuroendocrine, and neurotranscriptomic responses to anxiolytic and anxiogenic drugs are similar between zebrafish and mammals [19,23-26].

Many previous studies have documented behavioral, neuroendocrine, and neural bases of different coping styles but we are only beginning to understand the neurogenomic contributions to coping style variation [1,2,17,27,28]. The goal of this study is to characterize neurogenomic profiles that are associated with proactive and reactive stress coping styles. Rather than focusing solely on well-studied molecular pathways associated with the stress response, we aimed to identify other candidates in an unbiased manner by comparing neural transcriptomes through RNA-sequencing. Another objective was to understand the relationship of individual variation of baseline gene expression levels with variation in behavioral coping styles. We identify a core set of genes whose expression patterns are associated with proactive and reactive stress coping styles at both an individual and gene network level.

## Results

### Behavioral differences between lines and sexes

**Fig. 1:**
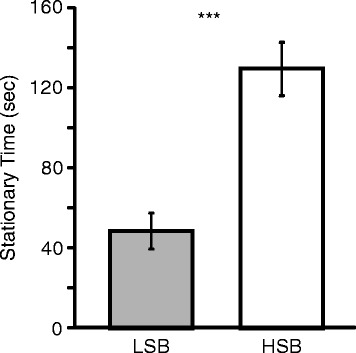
Stationary time in each line. LSB and HSB lines are gray and white fill, respectively. ***, p < 0.001

There was a significant main effect of line on stationary time (F = 27.766, p_one-tail_ = 3.5 * 10^−7^). The HSB line displayed significantly more time stationary than the LSB line (t = −5.086, p_one-tail_ = 7.6 * 10^−7^, Fig. 1) when controlling for sex. There was also a significant main effect of sex on stationary time (F = 8.382, p_one-tail_ = 0.002).Within both lines, females spent significantly more time stationary than males (LSB: t = 1.883, p_one-tail_ = 0.033; HSB, t = 2.22, p_one-tail_ = 0.015). There was no line x sex interaction effect on stationary time (F = 0.595, p = 0.442).

### Neurotranscriptome differences between lines

**Fig. 2:**
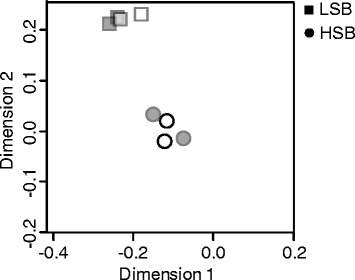
Multidimensional scaling plot of all genes for each biological replicate. Square and circle symbols are LSB and HSB lines, respectively. Filled and open symbols are male and female samples, respectively

Through RNA-sequencing we obtained on average 50 million reads for each biological replicate. For the protein coding genes in the zebrafish genome (Zv9, release 71), 14,867 genes passed default EdgeR filters. Multidimensional scaling plot analysis clearly revealed that the samples clustered together by line (Fig. 2). Of the genes analyzed, 1953 genes showed significant differences in basal transcript abundances between the HSB and LSB lines (Additional file 1: Table S1). Of these differentially expressed genes, 974 and 979 genes showed line-bias in the HSB and LSB lines, respectively. We subsequently measured eight genes (*comta*, *gabbr1a*, *gapdh*, *hsd11b2*, *oxtl*, *msmo1*, *prodha*, and *sell*) through qRT-PCR for validation of the RNA-sequencing results. All of the genes except for *gapdh* showed expression patterns consistent with our RNA-sequencing results (Additional file 2: Fig. S1,Additional file 3: Table S2). More specifically, after controlling for sex differences *comta* (F = 8.475, p_one-tail_ = 0.016), *prodha* (F = 14.357, p_one-tail_ = 0.006) and *sell* (F = 5.978, p_one-tail_ = 0.029) showed significantly higher expression in LSB than HSB. Genes with higher expression in HSB relative to LSB were *gabbr1a* (F = 18.988, p_one-tail_ = 0.003), hsd11b2 (F = 14.238, p_one-tail_ = 0.006), oxtl (F = 11.934, p_one-tail_ = 0.009), and *msmo1* (F = 24.864, p_one-tail_ = 0.002).

Gene ontology analyses showed that a number of key cellular processes are differentially regulated between the lines (Additional file 4: Table S3). In particular, for all genes identified as differentially expressed (Additional file 1: Table S1), they are generally associated with inter- and intra-neuronal communication, translation, T cell mediated immunity, and oxidation-reduction processes. After accounting for direction of expression, there are a number of gene ontology terms overrepresented in each line. Genes with higher expression in the LSB line are generally associated with protein metabolic process (e.g. translation), regulation of the cell cycle, and ribosomal proteins (Additional file 4: Table S3). Interestingly, genes showing HSB biased expression are associated with synaptic transmission, response to gonadotropin and purine-containing compound, cell adhesion, and transmembrane transporter activity (Additional file 4: Table S3).

### Weighted Gene Coexpression Network Analyses

**Fig. 3:**
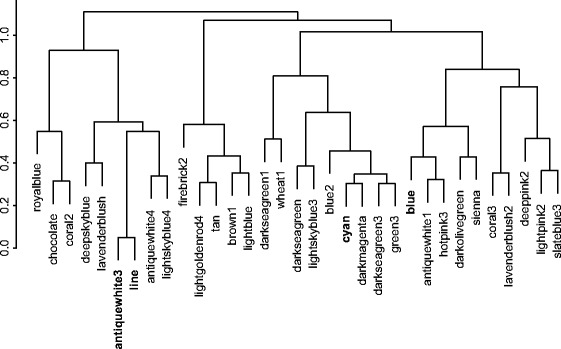
Hierarchical eigengene diagram of gene modules. All modules and dendrogram were obtained from WGCNA analysis. Modules in bold show a significant association with line differences (p < 0.05)

The use of weighted gene coexpression network analysis (WGCNA) in transcriptome studies serves to characterize gene coexpression networks and serves as an alternative method to identify genes associated with a trait (e.g. line differences) [29,30]. WGCNA analysis revealed that the neural transcriptome can be parceled into 30 modules of similarly coregulated genes (Fig. 3, Additional file 5: Table S4). Three modules were significantly associated with line differences (antiquewhite 3, p = 0.001; cyan, p = 0.03; blue, p = 0.01), but only the antiquewhite3 module remained significant after a Benjamini-Hochberg correction [31]. The antiquewhite3 module consists of 4788 genes (Additional file 5: Table S4). Gene ontology analysis shows that these genes are associated with metabolic, catalytic and lipid biosynthesis (Additional file 6: Table S5). To assess if genes more central to the antiquewhite3 network are more closely associated with line differences, we ran a Pearson correlation and observed that there was a significant and positive correlation (r = 0.89, p < 10^−200^) between gene significance for line and antiquewhte3 module membership.

### Core set of genes distinguishing coping styles

**Fig. 4:**
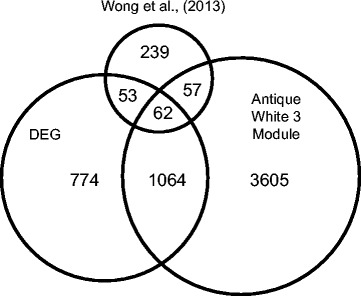
Venn diagram of genes identified to be associated with line differences. Genes comprise those found in edgeR analysis (DEG), WGCNA analysis (Antique White 3 Module), and anxiolytic drug treatment (Wong et al., (2013))

Unsurprisingly, transcriptome-wide analyses identified many genes that were differentially regulated by line (see above). To narrow down the list to a core set of genes associated with coping style, we focused on behavioral phenotype and genes which were similarly differentially expressed in this study (Additional file 1: Table S1) and our previous study assessing transcriptome effects of the anxiolytic drug fluoxetine in our HSB line fish [24]. We identified 115 genes that showed differential expression between comparison groups in the two studies (Fig. 4, Additional file 1: Table S1), and they showed significant congruence of expression (Binomial test, p = 2.8 * 10^−7^). More specifically, 85 of the genes (74 %) showed a consistent direction of expression differences across experiments (i.e. higher in LSB for current experiment and also higher in LSB-like in previous experiment). Gene ontology analyses revealed that organic acid biosynthetic process (GO:0016053, p = 0.0329), carboxylic acid biosynthetic process (GO:0046394, p = 0.0329), fatty acid metabolic process (GO:0006631, p = 0.0127) and fatty acid biosynthetic process (GO:0006633, p = 0.0472) terms were significantly overrepresented.

**Fig. 5:**
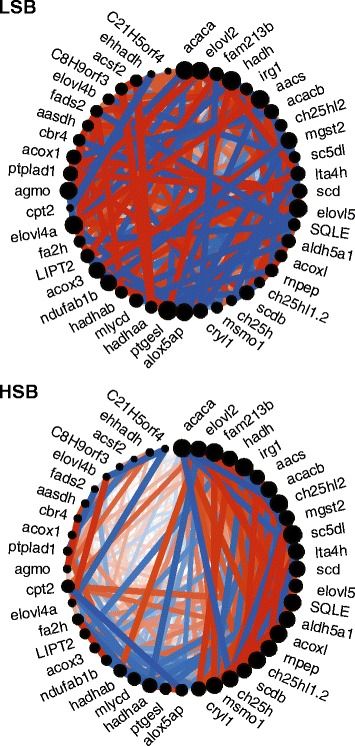
Fatty acid metabolic process gene coexpression network. Genes associated with fatty acid metabolic process showed low preservation in direction of correlation (color, red = r > 0, blue = r < 0), correlation coefficient (thickness = | r |), and network centrality (diameter of black circle) between (A) LSB and (B) HSB lines

Using a different analysis method, WGCNA, we identified genes in the antiquewhite3 module that were significantly associated with line differences (see above). To further narrow down the list of core genes associated with coping style, we compared the overlap of the gene lists generated by two different analysis methods in this study and our previous study [24]. We identified 62 genes that showed a common occurrence in all three lists (Fig. 4, Additional file 1: Table S1). These genes were associated with many organic acid biosynthetic processes including fatty acid metabolism (Table 1). Of note, WGCNA analyses demonstrated that genes associated with the GO term organic biosynthetic process and fatty acid metabolic process have low preservation between the HSB and LSB lines (Fatty acid metabolic process: Zsummary = 1.96, Fig. 5; Organic acid biosynethic process: Zsummary = 0.75, Additional file 7: Fig. S2).

**Table 1:** Gene ontology analysis of 62 genes found associated with coping style differences across analysis methods and experiments

Gene Ontology			
Category	Term	ID	p-value
BP	monocarboxylic acid metabolic process	GO:0032787	3.01E-03
BP	small molecule biosynthetic process	GO:0044283	4.06E-02
BP	organic acid biosynthetic process	GO:0016053	1.85E-02
BP	carboxylic acid biosynthetic process	GO:0046394	1.85E-02
BP	monocarboxylic acid biosynthetic process	GO:0072330	1.11E-02
BP	cellular lipid metabolic process	GO:0044255	3.95E-03
BP	fatty acid metabolic process	GO:0006631	5.71E-04
BP	very long-chain fatty acid metabolic process	GO:0000038	4.98E-02
BP	lipid biosynthetic process	GO:0008610	4.03E-03
BP	fatty acid biosynthetic process	GO:0006633	3.86E-03
BP	very long-chain fatty acid biosynthetic process	GO:0042761	4.98E-02

BP, Biological Process

### Individual variation in gene expression and behavior

To more directly assess the precise nature of the relationship between gene expression and behavioral stress coping styles, we examined whether individual variation in stationary behavior is related to variation in gene expression (*comta*, *gabbr1a*, *gapdh*, *hsd11b2*, *oxtl*, *msmo1*, *prodha*, and *sell*) through qRT-PCR of independent biological samples. There was no significant correlation between expression of any gene and stationary behavior exhibited by individual fish across both lines (*χ*2 < 1.7, p > 0.19). We observed, however, line-specific correlations between stationary time and presumed baseline gene expression levels (Additional file 8: Table S6). In the LSB line, there was a significant positive correlation between stationary time and the expression of *msmo1* and *hsd11b2*, but a negative correlation with *gabbr1a* (Additional file 8: Table S6). The HSB line, however, showed a different pattern such that there was a significant positive correlation in *oxtl* expression but a negative relationship with *gapdh* and stationary time (Additional file 8: Table S6).

## Discussion

Coping with stress involves the modulation of a variety of neural and physiological processes in the brain, which results in behavioral displays. Exploratory activity in novel or stressful environments is a distinguishing characteristic between reactive and proactive coping styles [1,2]. The HSB line showed a significantly higher duration of stationary time (i.e. time frozen) than the LSB line (Fig. 1) in the open field test, which is consistent with previous studies from this laboratory [19,32]. Higher exploratory activity in the open field test and lower freezing time in other anxiety-related behavioral paradigms is indicative of an animal that is proactively coping with stress (i.e. less anxious [1,2,27]). We have also previously shown that behavioral differences between the HSB and LSB lines are consistent over a variety of other behavioral paradigms [19] including the open field test and stationary time measurement used in the current study. Thus, our behavioral results suggest our lines differ in stress coping styles and do not represent a selection artifact on only general activity levels. It is noteworthy that many previous studies on anxiety-like behaviors and stress coping have used selection lines or strains in rodents and teleosts that are highly inbred [26,33-38]. Consequently there is likely a loss of phenotypic and genetic variation compared to individuals in the wild [1,39,40] including zebrafish [41], which may hinder detection of naturally occurring genetic mechanisms. Our zebrafish lines originated from approximately 200 wild caught individuals and were bidirectionally selected for differences in stationary behavior. Given that the zebrafish used in this study are only six and seven generations removed from the wild, it creates opportunities to identify genes associated with stress coping styles from a genetic background that is more similar to natural genetic variation in the wild.

Studies have shown that stress coping styles (i.e. anxiety-related behavioral responses) differ across strains despite being reared in similar conditions and exposed to the same testing paradigm, suggesting that genetic variation appears to play a key role in coping with stress. We identified 1953 genes that are differentially expressed between lines. Our results are consistent with previous studies documenting the involvement of GABAergic [42-44], nonapeptides [45,46], and glucocorticoid [47-51] neurotransmitter and molecular pathways in physiological and behavioral responses to stress. It should be emphasized that our fish did not experience a stressor prior to tissue collection for the RNA-sequencing results. This likely explains why we did not detect more differences in genes associated with the stress response between the lines. Regardless, of the genes showing significant differential expression by line, they support our behavioral results that the HSB and LSB lines are exhibiting the reactive and proactive stress-coping styles, respectively.

In addition to demonstrating the involvement of several well-documented molecular pathways associated with stress, we simultaneously assessed the association with other pathways in an unbiased manner. Using a gene ontology analysis on all 1953 differentially expressed protein-coding genes indicated that a variety of biological, molecular, and cellular properties are involved in distinguishing the coping styles (Additional file 4: Table S3). Of particular note is the overrepresentation of genes associated with the immune response and translation-related processes. The influences of cortisol and stress on the immune system are well documented [52]. As reactive coping style individuals are generally characterized by having a more pronounced endocrine stress response [1], we predict that the differences in genes associated with immunity are a consequence rather than a causal mechanism of variation in stress coping. One noteworthy result includes that many differentially expressed genes were associated with the processes of translation and, synaptic activity and plasticity. Previous studies have shown that synaptic plasticity is important for learning and glucocorticoid-mediated increases in protein synthesis in the brain facilitates memory [53,54]. Proactive coping style individuals rely heavily on previously ingrained memories (e.g. low behavioral flexibility) whereas reactive coping style individuals (e.g. high behavioral flexibility) will quickly learn and adapt to an environment when exposed to a stressor [27]. We observed that the LSB line (e.g. proactive) has higher basal expression of genes associated with protein synthesis and the HSB line (e.g. reactive) has higher expression of genes associated with synaptic plasticity and activity. We believe these results represent, in part, the molecular predispositions that facilitate the hypothesized differences in learning and memory between the HSB and LSB lines.

The LSB and HSB lines also showed differences in expression for genes associated with metabolic processes (e.g. oxidation-reduction, fatty acid metabolic and organic acid biosynthetic process, Additional file 4: Table S3). This is confirmed using WGCNA as an alternative analysis method to identify genes associated with line differences. The antiquewhite3 module showed overrepresentation of genes associated with small molecule and lipid metabolism (Additional file 6: Table S5). Furthermore, comparing across different analysis methods and experiments showed that the overlapping 62 gene set is associated with fatty acid metabolic activity (Additional file 1: Table S1). Changes in oxidation-reduction chemical processes are characteristic of oxidative stress [55]. Handling oxidative stress in the brain is a relatively recently described mechanism associated with anxiety and stress coping [55-57]. In particular glyoxalase, glutathione peroxidase and other genes in the oxidative stress pathway have been strongly linked to anxiety [58]. In this study, glyoxalase 1 and glutathione peroxidase showed significant differences in expression between our zebrafish lines (Additional File 1: Table S1). Unexpectedly, we observed glyoxalase 1 expression patterns to be opposite of what was documented in other studies, but other molecular mechanisms may be compensating [56]. Furthermore one consequence of oxidative stress is the breakdown of lipids (fatty acid metabolism – lipid peroxidation [59]). While the current study did not directly measure reactive oxygen species or lipid peroxidation products, we did observe changes in the expression of genes associated with lipid and fatty acid metabolic activity between the lines. It is plausible that the observed regulation of genes linked to lipid breakdown or synthesis could be due to oxidative stress. Taken together, these findings suggest that the HSB and LSB lines may differ in their oxidative stress load capacities. We hypothesize that possessing a proactive or reactive stress coping style may be due to the brain’s capability to handle oxidative stress.

Proactive and reactive stress coping styles are two qualitatively different responses to stressors observed in a variety of species. In addition to a relative bimodal distribution of coping styles in a population, there is also between-individual variation in the magnitude of the stress response within a coping style [1,5]. The genomic contribution to population and individual level variation in stress coping style is only beginning to be characterized (see [6,20,24,60] for zebrafish examples). Previous studies have identified genes associated with a particular coping style, but how these genes contribute to population or individual level variation is unknown (but see [6]). Through comparing data generated in the current as well as our previous pharmacological study, we identified a core set of 115 genes - whose functions are associated with fatty acid and metabolic and organic acid biosynthetic processes - that contribute to line level differences in stress coping styles. Intriguingly, the genes comprising fatty acid metabolic and organic acid biosynthetic process gene ontology terms showed low gene network preservation between lines (Fig. 5, Additional file 7: Fig. S2). This suggests that there is line specific gene coregulation of these two processes. Specifically, for both fatty acid metabolic and organic acid biosynthetic processes, the HSB line (reactive coping style) showed reduced coregulation across approximately half of the genes. Variation in coregulation of these genes might be a mechanism contributing to the approximate bimodal distribution of coping styles in a species. Variation in a variety of other behaviors has been attributed to changes in the coregulation of suites of genes [61-65]. Future studies should explore more precisely how changes in these gene networks modulate stress coping behavior.

To assess potential mechanisms contributing to inter-individual variation in stress coping, we examined the relationship between the expression of select genes and the amount of stationary behavior within each line. We selected eight genes (*comta*, *gabbr1a*, *gapdh*, *hsd11b2*, *oxtl*, *msmo1*, *prodh1a*, and *sell*) because they were differentially expressed between the lines or were previously associated with stress or anxiety-related behaviors in other studies [20,44,47,66,67]. Within each line, many genes showed no significant correlation between expression and stationary time, potentially because these genes have threshold effects on stationary behavior. The LSB fish showed a significant correlation between expression of *gabbr1a*, *msmo1*, and *hsd11b2* and stationary behavior. Surprisingly, *gabbr1a*, *msmo1*, and *hsd11b2* showed no significant correlation in the HSB line but *oxtl* and *gapdh* were significantly correlated with stationary time. Although we cannot determine the direction of causality from our study, we expect that the expression of the five genes can be used to predict the behavioral display. Given that many immediate early genes (class of genes encoding transcription factors that are rapidly transcribed with cellular activity; none of the eight genes analyzed are classified as immediate early genes) show peak transcript levels at 30 min. of stimulus exposure [68,69], our 5 min. behavioral trial likely does not give sufficient time for large changes in gene expression. Hence, we are likely measuring baseline expression levels of each gene and these levels can predict the amount of stationary time in an open field test (i.e. magnitude of the behavioral stress response). Overall, our results suggest that the variation in the behavioral stress response may be controlled by different molecular mechanisms within each line.

To our knowledge, only one other study has explored mechanisms of stress coping in zebrafish at the genomic scale [6]. We identified 215 genes that were similarly differentially regulated between our study and Rey et al. (2013). This, however, is not a greater number of common differentially regulated genes than expected by chance (hypergeometric test, p = 0.6). It is possible that we are observing independent molecular mechanisms that contribute to the same behavioral display. As our study utilized recently wild-derived selectively bred lines whereas Rey et al. behaviorally screened a commercial population to identify proactive and reactive stress coping styles, it suggests that different genetic architectures can lead to the same phenotype. Although it is outside the scope of the current study to explore across other taxa, our study provides a basis for such meta-analyses. For example, evidence from both the current and our previous pharmaceutical manipulation studies suggest that fatty acid metabolic processes may be key mechanisms for stress coping. Future studies should examine the role of fatty acids in stress coping in other species.

## Conclusions

The behavioral and physiological responses to stressors vary within a species, but nonetheless are considered adaptive in a natural context. The proactive and reactive stress coping styles are found in a diverse range of animal taxa suggesting stress coping styles are highly conserved. It is uncertain if the underlying proximate mechanisms are similarly conserved or if independent mechanisms can generate the same phenotype. In this study we i) identify genes associated with stress coping styles, ii) characterize differences in gene coexpression networks and iii) characterize differences in line-specific regulation of gene expression related to the magnitude of behavioral displays. Differences in basal whole brain neurotranscriptomic states between lines might explain the observed behavioral differences. Individuals from the proactive coping style line (LSB) had increased expression of genes associated with translation (e.g. amino acid metabolism and ribosomal proteins). Reactive coping style individuals (HSB) showed upregulation of genes linked to inter- and intra-neuronal communication and response to gonadotropin stimulus. Ultimately, through comparisons across studies from our laboratory, we identify a core set of 115 genes that differentiates coping styles. This core set of genes shows a significant congruence in the direction of expression between the two studies (i.e. elevated expression in the proactive coping style line (LSB) is similarly elevated in the pharmacologically induced proactive coping style (anxiolytic-treated fish [24])), which suggests these genes are not artifacts of our selection procedure or drug effects. These genes represent fatty acid metabolic and organic acid biosynthetic processes, which may be key processes mediating differences in stress coping styles. These cellular processes may be more broadly linked to oxidative stress and neurometabolism, two processes that warrant further investigation on their precise roles in coping with stress.

## Methods

In this study we conducted two separate experiments. The first experiment’s goal was to identify differentially expressed genes and characterize the neurogenomic states between lines of zebrafish that we have previously shown to differ in stress coping styles [19]. More specifically for each line (LSB and HSB) we generated one cohort where we behaviorally tested 56 fish to confirm behavioral differences between our lines. Three weeks later we collected whole-brain tissue from 40 different fish of the same cohort that were immediately sacrificed after capturing from their home tank and did not undergo a behavioral test (see below for exception). These subjects underwent RNA-sequencing. In the second experiment, the goal was to further elucidate the direction of relationship between select genes (identified in first experiment) and stationary behavior. We behaviorally tested 18 additional fish from each line and extracted whole-brain tissue immediately following a behavioral trial. Using quantitative reverse-transcriptase PCR, we subsequently assessed the correlation between individual variation of gene expression and stationary time. See below for additional methodological descriptions.

### Behavioral assay

To quantify behavioral stress-coping styles, we utilized the open field test adapted for zebrafish six generations removed from the wild progenitor fish and used previously in our laboratory [19,32,70]. All fish were maintained in mixed sex 100-liter tanks on a recirculating filtration system at 28 °C with a 12:12 light dark cycle and fed daily. Briefly, we exposed individual male and female fish from the HSB (n = 56) and LSB (n = 56) lines to a 30 x 30 x 10 cm (width x length x height) arena filled with four liters of aquarium system water (water used to house fish). For the 5-min. trial we recorded the amount of time spent stationary (moving less than 0.1 cm/s) using automated software (TopScan Lite, Reston, VA, USA). For 18 fish of each line, which were seven generations removed from the wild progenitor fish, we immediately sacrificed after the behavioral assay and prepared for quantitative reverse transcriptase PCR analysis (see below). We assessed differences in stationary time using a general linear model with sex and line as cofactors (SPSS version 20). From our previous study, we predicted that the HSB and females will have higher stationary times than the LSB and males, respectively. Consequently, we assessed statistical significance using one-tail p-values. All procedures and protocols in this study were approved by the North Carolina State University Institutional Animal Care and Use Committee.

### Neurotranscriptome quantification by RNA-sequencing

We used HSB and LSB individuals six generations removed from the wild. All individuals (n = 40 for each line) were 17 weeks post-fertilization and sexually mature. Fish were removed from their home tanks and quickly sacrificed between 09:00 – 12:00. Sex and maturity were confirmed by visual inspection of gonads. Fish were quickly caught from their home tanks, immediately decapitated, and the brains removed in under 3 min. following decapitation. Brains were stored in RNAlater (Ambion, Austin TX) at 4 °C overnight and then stored at −80 °C until RNA extraction. Due to limited numbers of fish in the HSB and LSB lines, 15 of the individuals we sampled from each of these lines had undergone behavioral testing three weeks prior (see above).

We used our previously established protocols for RNA extraction and RNA-sequencing [24,70]. Briefly, we extracted RNA from 80 individuals (40 individuals of each line) using RNeasy Plus Mini Kit (Qiagen). As the goal of this part of the experiment was to assess a general effect of coping style on the transcriptomes, for each line we pooled one microgram of total RNA from 10 same sex individuals into one biological replicate. This resulted in four biological replicates for each line (two each for males and females). RNA quality was assessed with an Agilent 2100 Bioanalyzer (Agilent) and all samples had RNA integrity numbers (RIN) above 8.0. We followed the manufacturer’s protocol for cDNA library preparation (TruSeq RNA Sample Prep V2, Illumina) and submitted our samples to the Genomic Sciences Laboratory at North Carolina State University for 72 bp single-end RNA sequencing (Illumina GAIIx). We combined reads across all lanes that passed default quality control filters, which resulted in an average of 50 million reads per biological replicate. This data is accessible through NCBI’s Gene Expression Omnibus [GEO: GSE61108]. We aligned the reads to the *Danio rerio* genome (assembly Zv9 [15], release 71) using GSNAP [71] with default parameters. We used HTSeq to quantify the number of reads aligned to each gene using the “union” mode. We employed a two-factor design using EdgeR [72] to assess differential expression of protein-coding genes between the lines with sex as a cofactor. We used gProfiler [73,74] to determine significantly over-enriched gene ontology (GO) terms. We utilized the default false discovery rate (FDR) corrections in both EdgeR and gProfiler. Statistical significance was defined as p_FDR-corrected_ < 0.05.

### Gene Coexpression Network Analysis

Using normalized expression counts from all the genes that underwent differential expression analysis in edgeR, we characterized gene expression network dynamics. The analyses utilized an open-source software, weighted gene co-expression network analysis (WGCNA [29]). WGCNA clusters together highly correlated genes into modules (arbitrarily given a color named by the software package), which can then be used to assess a variety of attributes (see [29] and references within for full details). Our goal was to identify modules associated with line differences. WGCNA analysis revealed that one of the LSB line female biological replicates was an outlier and we removed that sample from all WGCNA analyses. To identify modules associated with line, we ran WGCNA using the seven biological replicate pools. Subsequently, within modules that passed FDR correction, we assessed the relationship between gene significance for line and module membership. Module membership represents the correlation of the module eigengene and the gene expression profile and is used as a proxy for measuring how central the gene is within the module (see [29] for more details).

We ran separate WGCNA analyses for each line to assess the preservation of genes assigned to the gene ontology terms fatty acid metabolic process (GO: 0006331) and organic acid biosynthetic process (GO: 00016053) between HSB and LSB. We selected these gene ontology terms because they were significantly over-enriched from a shared gene list identified by comparing across the current and our previous experiment [24] and were parent terms. Analysis and visualization of the preservation of these genes between the lines followed an established protocol [30,70]. Briefly, we adjusted soft-threshold (β) values to ensure an approximate scale-free topology [75], set the minimum module size to 30, dynamic tree cut height to 0.3 to ensure a larger number of genes in each module to assess intramodule dynamics, and used the default parameters for all other WGCNA settings. Module preservation statistics across line were conducted and defined as in [30]: Preservation Z-Summary scores greater than 10, between 10 and 2, and less than 2 are designated as strongly, moderately, and weakly preserved. Preservation Z-Summary is a composite summary statistic that includes measures of density and connectivity between networks and is used to measure the preservation of network properties within a module or set of genes between two networks (see [30] for more details).

### Identifying genes associated with stress-coping style

To identify a core set of genes associated with stress-coping style, we compared differentially expressed gene lists from different analysis methods in the current study with those from our previous pharmacological study [24]. Briefly, in our pharmacological study we chronically administered an anxiolytic drug (fluoxetine, a selective serotonin reuptake inhibitor) to the HSB (e.g. reactive) line and measured changes in behavior and gene expression profiles. The drug effectively induced the alternative behavioral stress coping style in the HSB line (behaved similarly to LSB line, i.e. LSB (or proactive)-like). We used genes identified as significantly differentially expressed between lines using edgeR, genes that were in the antiquewhite3 module, and genes that were differentially expressed with an anxiolytic drug treatment. For genes that overlapped across the two experiments’ lists, we used a binomial test to assess if genes up-regulated in the LSB line were also up-regulated with anxiolytic drug treatment in [24]. As our pharmacological study administered a drug that is a selective serotonin reuptake inhibitor, we acknowledge that any identified overlapping genes may be limited to those modulated by the serotonergic system.

### Quantitative reverse-transcriptase PCR

We performed quantitative reverse transcriptase PCR (qRT-PCR) on i) pooled samples used for RNA-sequencing for technical validation and ii) independent biological samples to assess relationship between behavior and gene expression. We selected the following genes because they show line differences in zebrafish from our RNA-sequencing results or are associated with stress and anxiety-related behaviors in other species [20,44,47,66,67]: *comta* (catechol-o-methyltransferase a), *gabbr1a* (gamma-aminobutyric acid (GABA) B receptor, 1a), *oxtl* (oxytocin-like), *msmo1* (methylsterol monooxygenase 1), *gapdh* (glyceraldehyde-3-phosphate dehydrogenase), *hsd11b2* (hydroxysteroid 11-beta dehydrogenase 2), *prodha* (proline dehydrogenase (oxidase) 1a), *sell* (Selenoprotein L). Fish in ii) that underwent a behavioral assay were immediately sacrificed after open field testing (see above). Preparation, execution, and analysis of the qRT-PCR followed methods described previously [24,70]. Briefly, we homogenized tissue in Trizol (Invitrogen) and extracted the RNA through column filtration (RNeasy Plus Mini Kit, Qiagen). RNA (1 μg) was subsequently converted to cDNA (SuperScript III First-Strand Synthesis System for qRT-PCR, Invitrogen) and purified (Amicon Ultra −0.5 mL 30 K Centrifugal Filters, Millipore). We ran qRT-PCR reactions on an ABI 7900HT Fast Real-Time PCR system (Applied Biosystems) using SYBR Select (Applied Biosystems). Primers either spanned exon-exon junctions or the amplicon spanned two exons with an included intron region over 1 kilobase. Each sample was run in triplicate (see Additional file 9: Table S7 for primer sequences, amplicon lengths, and qRT-PCR reaction parameters). We normalized gene expression to an endogenous reference gene (*ef1a*). Expression of *ef1a* has been shown to be stable across sex, age, and chemical treatment in zebrafish [76]. We used a general linear model with sex as a cofactor to assess differences in gene expression between the lines. We predicted that qRT-PCR patterns would follow those seen in the RNA-sequencing analysis and assess statistical significance using one-tailed p-values. We examined relationships between gene expression and stationary behavior using a generalized linear model with sex as a cofactor and gene expression as covariates. We determined significance with two-tailed p-values. Statistical analyses were performed in SPSS (version 20).

### Availability of supporting data

The data sets supporting the results of this article are included within the article (and its additional files). Data is also accessible through NCBI’s Gene Expression Omnibus [GEO:GSE61108].

## Additional files

Additional file 1: Table S1. Description of data: Quantification (counts per million) and statistical results of all genes that underwent edgeR differential expression analysis. Bold are genes that were also identified as differentially expressed with anxiolytic drug treatment [24]. Yellow cell color are genes associated with line differences by comparing different analysis methods (edgeR, WGCNA) and experiments (artificial selection, pharmacology). Additional file 2: Figure S1. Description of data: Technical validation of gene expression of select genes. Gene expression as measured by qRT-PCR of select genes. Expression is normalized by expression of endogenous reference control, *ef1a*. LSB and HSB are gray and white bars, respectively. *, p < 0.05; **, p < 0.01. Additional file 3: Table S2. Description of data: Statistical analysis of select genes for technical validation. General linear model included sex as a cofactor. Additional file 4: Table S3. Description of data: Gene ontology analysis of differentially expressed genes. Additional file 5: Table S4. Description of data: Module classification of recently wild-derived zebrafish transcriptomes. Additional file 6: Table S5. Description of data: Gene ontology analysis of genes in antiquewhite3 module. Additional file 7: Figure S2. Description of data: Organic acid biosynthetic process gene coexpression network. Genes associated with organic acid biosynthetic process showed *low* preservation in direction of correlation (color, red = r > 0, blue = r < 0), correlation coefficient (thickness = | r |), and network centrality (diameter of black circle) between LSB and HSB lines. Additional file 8: Table S6. Description of data: Correlation between gene expression and stationary behavior by line. Generalized linear model included sex as a cofactor and each gene’s expression as covariates. Additional file 9: Table S7. Description of data: qRT-PCR primer characteristics.
