# Supplementary figures and images for: Characterizing the neurotranscriptomic states in alternative stress coping styles
Source: BMC Genomics. 2015 Jun 2;16(1):425. doi: 10.1186/s12864-015-1626-x (PMC4450845; doi:10.1186/s12864-015-1626-x)

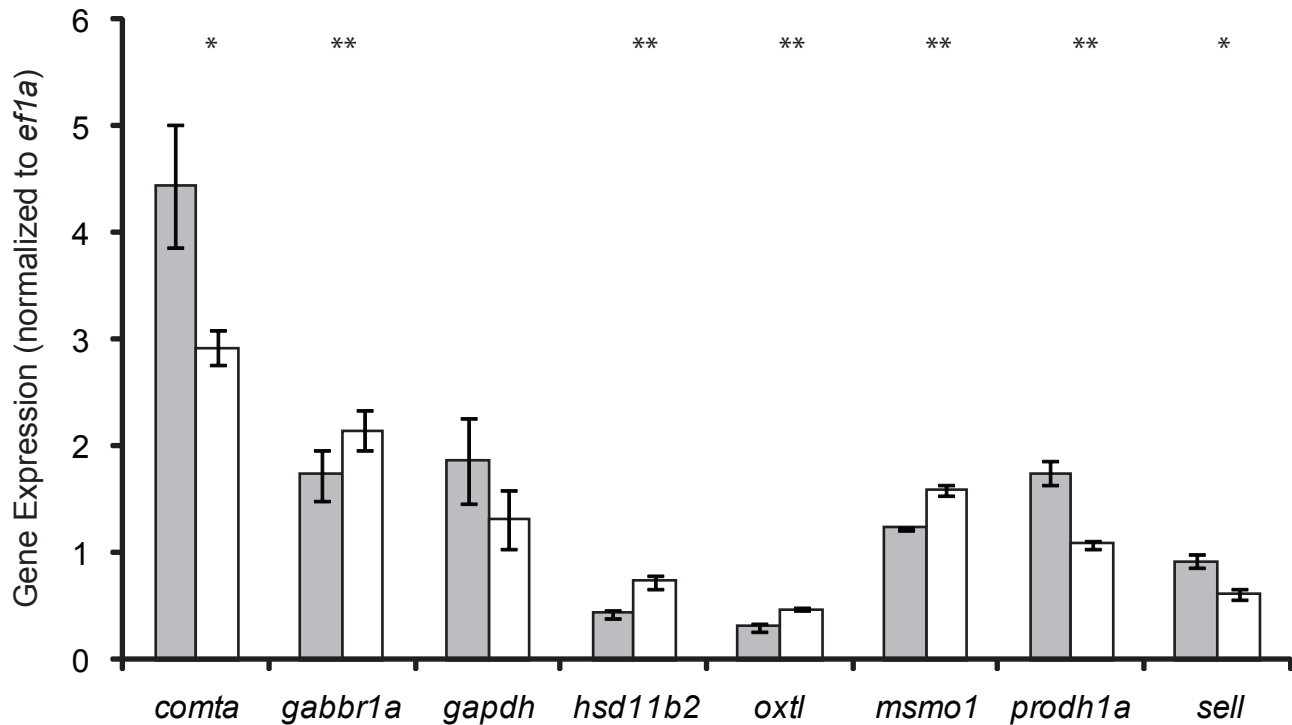

Supplement: Additional file 2: Figure S1. — Description of data: Technical validation of gene expression of select genes. Gene expression as measured by qRT-PCR of select genes. Expression is normalized by expression of endogenous reference control, ef1a. LSB and HSB are gray and white bars, respectively. *, p < 0.05; **, p < 0.01. [file 12864_2015_1626_MOESM2_ESM.pdf]

LSB

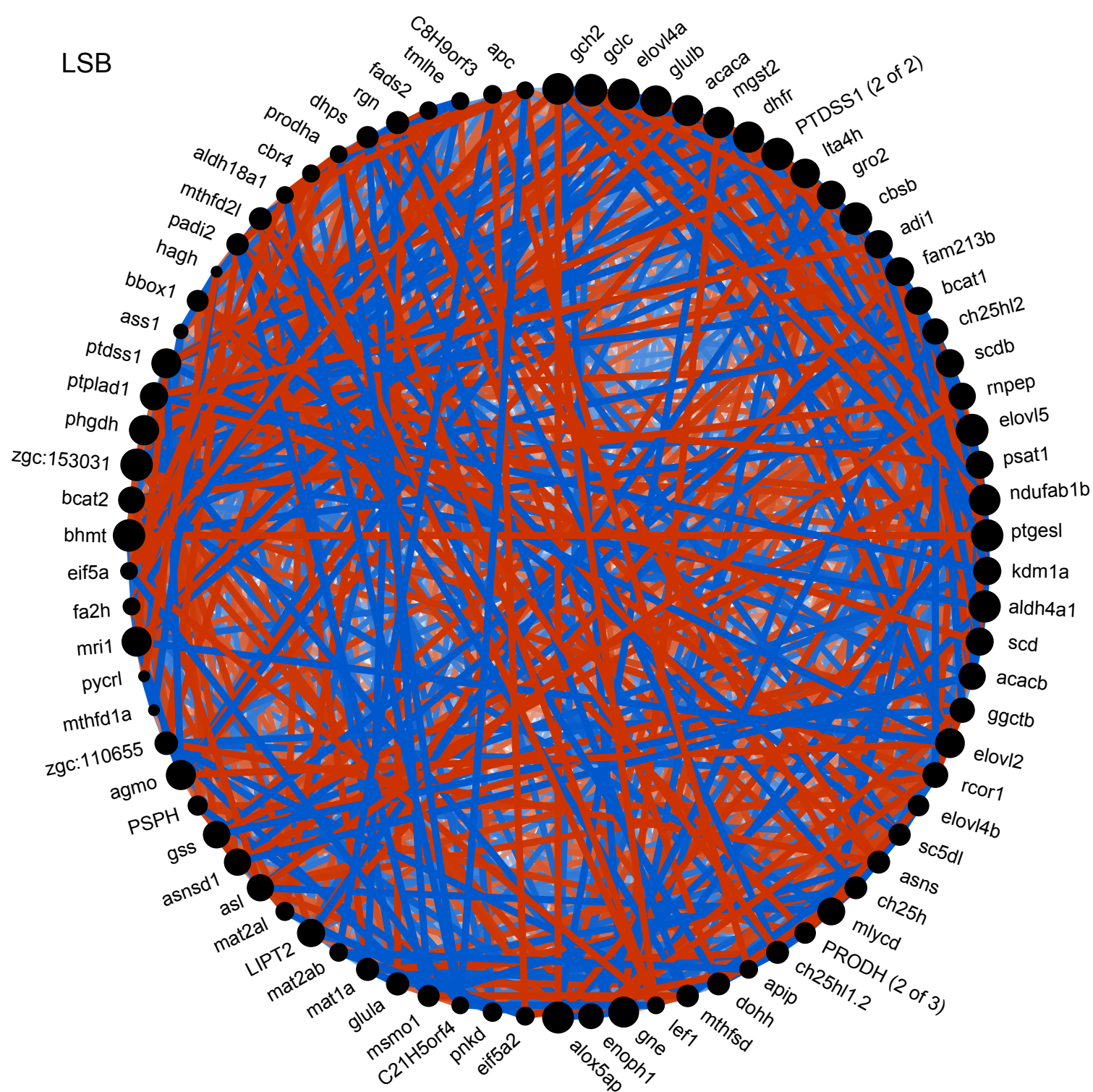

HSB

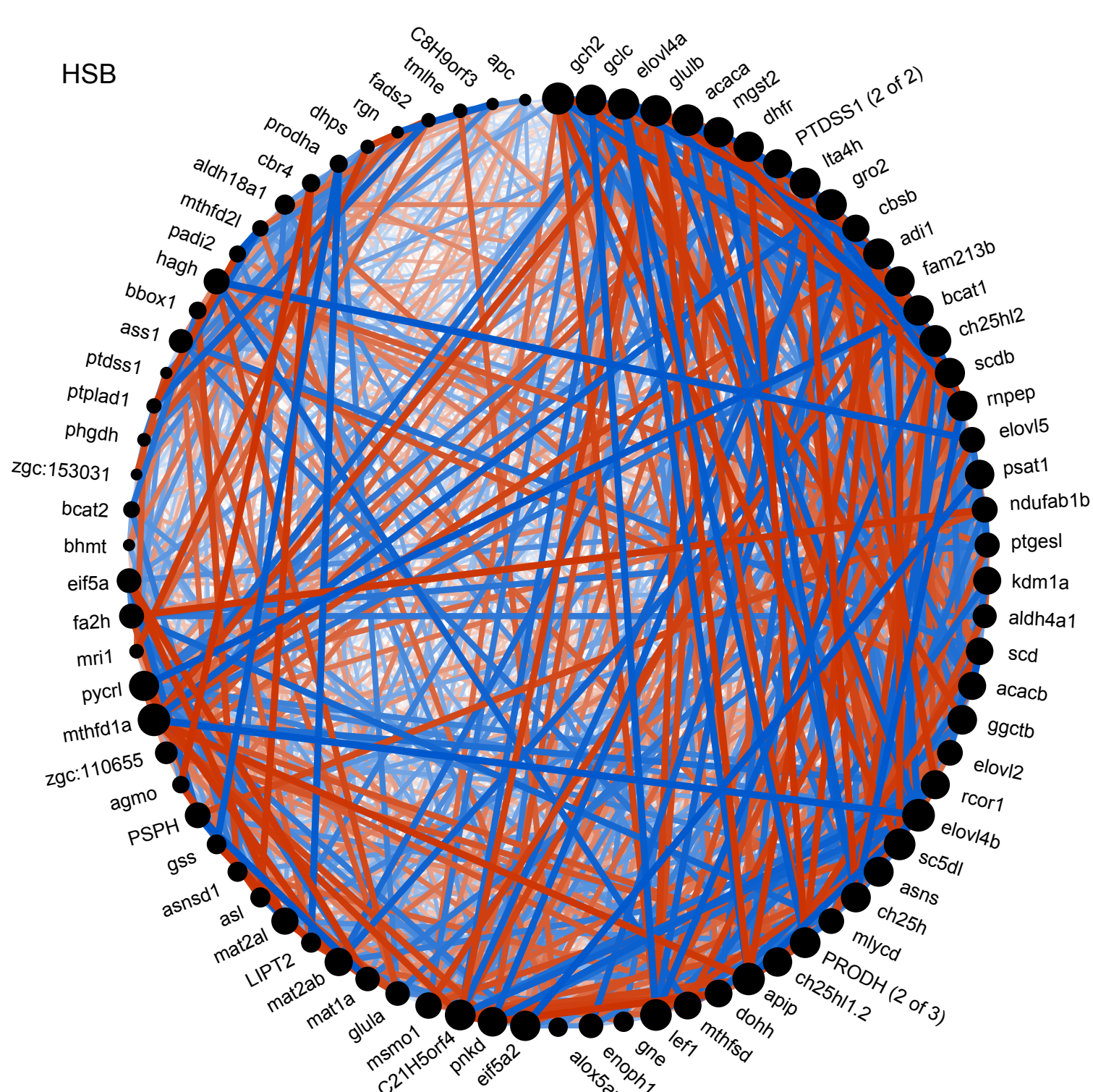

Supplement: Additional file 7: Figure S2. — Description of data: Organic acid biosynthetic process gene coexpression network. Genes associated with organic acid biosynthetic process showed low preservation in direction of correlation (color, red = r > 0, blue = r < 0), correlation coefficient (thickness = | r |), and network centrality (diameter of black circle) between LSB and HSB lines. [file 12864_2015_1626_MOESM7_ESM.pdf]
