# Supplementary material for: Characterizing the neurotranscriptomic states in alternative stress coping styles
Source: BMC Genomics. 2015 Jun 2;16(1):425. doi: 10.1186/s12864-015-1626-x (PMC4450845; doi:10.1186/s12864-015-1626-x)
Supplement: Additional file 3: Table S2. — Description of data: Statistical analysis of select genes for technical validation. General linear model included sex as a cofactor. [file 12864_2015_1626_MOESM3_ESM.pdf]

Additional Table 2. Gene Expression between the HSB and LSB lines

Gene Symbol	F	p-value $_{one\text{-}tail}$
COMTA	8.475	0.017
GABBR1A	18.988	0.004
GAPDH	0.823	0.273
HSD11B2	14.238	0.007
MSMO1	24.864	0.002
OXT	11.934	0.009
PRODH1A	14.357	0.007
SELL	5.978	0.029
