# Supplementary material for: Characterizing the neurotranscriptomic states in alternative stress coping styles
Source: BMC Genomics. 2015 Jun 2;16(1):425. doi: 10.1186/s12864-015-1626-x (PMC4450845; doi:10.1186/s12864-015-1626-x)
Supplement: Additional file 4: Table S3. — Description of data: Gene ontology analysis of differentially expressed genes. [file 12864_2015_1626_MOESM4_ESM.docx]

Additional Table 3. Gene ontology analysis of differentially expressed genes

| Gene Ontology | | | FDR corrected p-value | | |
| --- | --- | --- | --- | --- | --- |
| Category | Term | ID | DEG (n=1953) | LSB -biased (n= 979) | HSB -biased (n=974) |
| BP | deoxyribonucleoside diphosphate metabolic process | GO:0009186 | 2.55E-03 |  |  |
| BP | mitochondrion morphogenesis | GO:0070584 | 2.41E-02 |  |  |
| BP | T cell mediated immunity | GO:0002456 | 4.34E-02 |  |  |
| BP | T cell mediated cytotoxicity | GO:0001913 | 4.34E-02 |  |  |
| BP | regulation of T cell mediated immunity | GO:0002709 | 4.34E-02 |  |  |
| BP | regulation of T cell mediated cytotoxicity | GO:0001914 | 4.34E-02 |  |  |
| BP | positive regulation of adaptive immune response | GO:0002821 | 4.34E-02 |  |  |
| BP | positive regulation of adaptive immune response based on somatic recombination of immune receptors built from immunoglobulin superfamily domains | GO:0002824 | 4.34E-02 |  |  |
| BP | positive regulation of T cell mediated immunity | GO:0002711 | 4.34E-02 |  |  |
| BP | positive regulation of T cell mediated cytotoxicity | GO:0001916 | 4.34E-02 |  |  |
| BP | oxidation-reduction process | GO:0055114 | 2.21E-02 |  |  |
| BP | synaptic transmission | GO:0007268 | 3.78E-03 |  | 8.58E-04 |
| BP | translation | GO:0006412 | 1.95E-16 | 1.52E-32 |  |
| BP | translational elongation | GO:0006414 | 3.44E-02 | 8.02E-05 |  |
| BP | ribosomal small subunit biogenesis | GO:0042274 | 1.27E-04 | 4.21E-05 |  |
| BP | maturation of SSU-rRNA | GO:0030490 | 2.02E-02 | 4.19E-02 |  |
| BP | maturation of SSU-rRNA from tricistronic rRNA transcript (SSU-rRNA, 5.8S rRNA, LSU-rRNA) | GO:0000462 | 7.91E-03 | 2.15E-02 |  |
| BP | cellular protein metabolic process | GO:0044267 |  | 2.88E-03 |  |
| BP | ribonucleoprotein complex biogenesis | GO:0022613 |  | 2.00E-02 |  |
| BP | ribosome biogenesis | GO:0042254 |  | 1.49E-02 |  |
| BP | regulation of cell cycle | GO:0051726 |  | 3.42E-02 |  |
| BP | response to organophosphorus | GO:0046683 |  |  | 2.47E-03 |
| BP | response to purine-containing compound | GO:0014074 |  |  | 2.47E-03 |
| BP | response to cAMP | GO:0051591 |  |  | 2.47E-03 |
| BP | cellular response to cAMP | GO:0071320 |  |  | 2.47E-03 |
| BP | response to gonadotropin | GO:0034698 |  |  | 2.47E-03 |
| BP | cellular response to gonadotropin stimulus | GO:0071371 |  |  | 2.47E-03 |
| BP | biological adhesion | GO:0022610 |  |  | 1.37E-02 |
| BP | cell adhesion | GO:0007155 |  |  | 1.37E-02 |
| CC | MHC class I protein complex | GO:0042612 | 3.05E-02 |  |  |
| CC | non-membrane-bounded organelle | GO:0043228 | 2.66E-07 | 3.45E-15 |  |
| CC | intracellular non-membrane-bounded organelle | GO:0043232 | 2.66E-07 |  |  |
| CC | macromolecular complex | GO:0032991 | 4.30E-05 |  |  |
| CC | cytoplasm | GO:0005737 | 2.70E-08 | 1.27E-14 |  |
| CC | cytoplasmic part | GO:0044444 | 4.00E-08 | 6.11E-16 |  |
| CC | cytosol | GO:0005829 | 2.24E-02 | 7.71E-03 |  |
| CC | cytosolic part | GO:0044445 | 5.48E-03 | 2.22E-04 |  |
| CC | ribonucleoprotein complex | GO:0030529 | 2.74E-20 | 5.75E-35 |  |
| CC | ribosome | GO:0005840 | 1.67E-26 | 5.18E-42 |  |
| CC | cytosolic ribosome | GO:0022626 | 2.89E-06 | 1.06E-08 |  |
| CC | ribosomal subunit | GO:0044391 | 1.15E-09 | 1.11E-14 |  |
| CC | small ribosomal subunit | GO:0015935 | 1.36E-09 | 1.04E-13 |  |
| CC | cytosolic small ribosomal subunit | GO:0022627 | 3.50E-05 | 2.39E-07 |  |
| CC | organelle | GO:0043226 |  | 8.92E-05 |  |
| CC | macromolecular complex | GO:0032991 |  | 1.83E-16 |  |
| CC | organelle part | GO:0044422 |  | 1.78E-05 |  |
| CC | intracellular | GO:0005622 |  | 2.96E-05 |  |
| CC | intracellular part | GO:0044424 |  | 5.25E-07 |  |
| CC | intracellular organelle | GO:0043229 |  | 1.58E-04 |  |
| CC | intracellular non-membrane-bounded organelle | GO:0043232 |  | 3.45E-15 |  |
| CC | intracellular organelle part | GO:0044446 |  | 3.82E-05 |  |
| MF | oxidoreductase activity | GO:0016491 | 1.31E-02 |  |  |
| MF | transferase activity, transferring amino-acyl groups | GO:0016755 | 8.40E-03 |  |  |
| MF | gamma-glutamylcyclotransferase activity | GO:0003839 | 4.37E-03 | 2.86E-02 |  |
| MF | iron ion binding | GO:0005506 | 6.35E-03 |  |  |
| MF | structural molecule activity | GO:0005198 | 2.06E-18 | 1.05E-29 |  |
| MF | structural constituent of ribosome | GO:0003735 | 1.21E-26 | 2.70E-41 |  |
| MF | peptide antigen binding | GO:0042605 | 4.34E-02 |  |  |
| MF | transmembrane transporter activity | GO:0022857 |  |  | 3.60E-02 |
| MF | secondary active transmembrane transporter activity | GO:0015291 |  |  | 4.47E-02 |

BP, Biological Process; MF, Molecular Function; DEG, all differentially expressed genes. Blank cells indicate p > 0.05.
