# Supplementary material for: Characterizing the neurotranscriptomic states in alternative stress coping styles
Source: BMC Genomics. 2015 Jun 2;16(1):425. doi: 10.1186/s12864-015-1626-x (PMC4450845; doi:10.1186/s12864-015-1626-x)
Supplement: Additional file 6: Table S5. — Description of data: Gene ontology analysis of genes in antiquewhite3 module. [file 12864_2015_1626_MOESM6_ESM.pdf]

Additional Table 5. Gene ontology analysis for genes in antiquewhite3 module

Gene Ontology			
Category	Term	ID	p-value
BP	phosphorus metabolic process	GO:0006793	2.13E-02
BP	phosphate-containing compound metabolic process	GO:0006796	1.54E-02
BP	organophosphate metabolic process	GO:0019637	2.02E-03
BP	single-organism metabolic process	GO:0044710	7.70E-03
BP	small molecule metabolic process	GO:0044281	1.07E-04
BP	nucleobase-containing small molecule metabolic process	GO:0055086	4.96E-02
BP	single-organism biosynthetic process	GO:0044711	1.15E-02
BP	small molecule biosynthetic process	GO:0044283	2.16E-02
BP	glycosyl compound metabolic process	GO:1901657	1.98E-02
BP	lipid biosynthetic process	GO:0008610	1.76E-02
BP	nucleoside metabolic process	GO:0009116	2.30E-02
BP	ribonucleoside metabolic process	GO:0009119	4.69E-02
BP	cellular lipid metabolic process	GO:0044255	2.35E-04
CC	intracellular part	GO:0044424	1.04E-02
CC	cytoplasm	GO:0005737	1.59E-04
MF	catalytic activity	GO:0003824	1.44E-04
