# Supplementary material for: Characterizing the neurotranscriptomic states in alternative stress coping styles
Source: BMC Genomics. 2015 Jun 2;16(1):425. doi: 10.1186/s12864-015-1626-x (PMC4450845; doi:10.1186/s12864-015-1626-x)
Supplement: Additional file 8: Table S6. — Description of data: Correlation between gene expression and stationary behavior by line. Generalized linear model included sex as a cofactor and each gene’s expression as covariates. [file 12864_2015_1626_MOESM8_ESM.pdf]

Additional Table 6. Correlation between individual variation of stationary time and presumed baseline gene expression by line.

Gene	LSB			HSB		
	B	χ^2	p-value	B	χ^2	p-value
comta	3.353	.946	0.331	2.428	.343	0.558
gabbr1a	-16.154	4.107	0.043	-4.773	.089	0.766
prodh1a	-17.792	3.462	0.063	-1.674	.006	0.940
sell	47.913	2.067	0.150	13.295	.061	0.804
msmo1	86.220	4.042	0.044	-67.851	3.218	0.073
oxtl	50.161	1.520	0.218	189.905	8.118	0.004
gapdh	.267	.161	0.689	-4.034	9.762	0.002
hsd11b2	78.812	23.913	1×10^{-6}	54.363	1.463	0.227
