# Supplementary material for: Characterizing the neurotranscriptomic states in alternative stress coping styles
Source: BMC Genomics. 2015 Jun 2;16(1):425. doi: 10.1186/s12864-015-1626-x (PMC4450845; doi:10.1186/s12864-015-1626-x)
Supplement: Additional file 9: Table S7. — Description of data: qRT-PCR primer characteristics. [file 12864_2015_1626_MOESM9_ESM.pdf]

Additional Table 7. qRT-PCR primer characteristics

Gene Symbol	Forward Primer	Reverse Primer	Amplicon Length
COMTA	5'-TCTGGCACGATGTGGTCCAT-3'	5'-TCAAGATGCGCTGTGGTCGT-3'	72
EF1A	5'-CCTCTTGGTCGCTTTGC-3'	5'-GGTGTGATTGAGGGAAATTCA-3'	150
HSD11B2	5'-GCCTATAAGACAGGGCAGAGCA-3'	5'-TTTGGGGACAAGCCCTGGAG-3'	80
GABBR1A	5'-CCCAGAGACGGAGGGATACG-3'	5'-CGGGCACATCATCAAGCATCT-3'	198
GAPDH	5'-TGGGGTGATGCAGGTGCTAC-3'	5'-ACCCTTAATGTGAGCAGAAGCCT-3'	84
MSMO1	5'-CCAGCCGGATAAACCAGAGACA-3'	5'-CGCAGATGAGCGGTAGCTGT-3'	95
OXT	5'-ATTCGACAGTGTATGCCGTG-3'	5'-TCACACGGAGAAGGGAGAAA-3'	146
PRODH1A	5'-GACGCAAGAAGAGGCCGAGA-3'	5'-GGTGATCCATACCGTGGCTCT-3'	80
SELL	5'-GAGAAAGACACTGCGAATGGATGTA-3'	5'-TACAGAACTTTGCCCCTGCTG-3'	94

qRT-PCR parameters (all genes): 2 min at 50°C, 2 min at 95°C, followed by 40 cycles at 15 sec 95°C and 1 min 60°C. Primer concentration was 5 pmol for COMTA, EF1A, GABBR1A, MSMO1, and OXT, and 1.5 pmol for HSD11B2, GAPDH, PRODH1A, and SELL.
